# Enteric Methane Emissions from Holstein Cows Grazing Kikuyu Grass in the Colombian High Tropics During Two Seasons

**DOI:** 10.3390/ani16111662

**Published:** 2026-05-29

**Authors:** Ligia Johana Jaimes Cruz, Karla Fernanda Molina Macias, Santiago Cadavid Henao, Mariano Eliecer Acosta Lobo, Wilmer Alfonso Cuervo Vivas, María Victoria Galeano Correa, Héctor Jairo Correa Cardona, José Eduardo Escobar Riomalo, Ángel Giraldo Mejía

**Affiliations:** 1 Faculty of Veterinary Medicine and Zootech, Institución Universitaria Visión de las Américas, Campus Medellín, Medellín 050001, Colombiamariano.acosta@uam.edu.co (M.E.A.L.);; 2 Department of Animal and Range Sciences, Montana State University, Bozeman, MT 59718, USA; wilmer.cuervovivas@montana.edu (W.A.C.V.); maria.galeano@student.montana.edu (M.V.G.C.); 3 Department of Animal Production, Universidad Nacional de Colombia, Campus Medellín, Medellín 050001, Colombia; hjcorreac@unal.edu.co (H.J.C.C.); agiraldom@unal.edu.co (Á.G.M.)

**Keywords:** sensors, spirometry mask, greenhouse gases, cattle

## Abstract

Enteric methane emissions (EMEs) from grazing dairy systems in tropical regions remain poorly quantified, increasing uncertainty in national greenhouse gas inventories. This study aimed to quantify EMEs using electronic spirometry masks (ESMs) in dairy cows in the Colombian high tropics during two precipitation seasons in two adjacent milk production management systems. Six cows of a high-milk-yield management system (HMYMS; >30 L/d) and six cows of a low-milk-yield management system (LMYMS; <15 L/d) grazing kikuyu grass (*Cenchrus clandestinus*) and supplemented with concentrate feed were monitored by EMEs, exhaled air volume, feed intake, milk yield and composition. Data were analyzed in a 2 × 2 factorial arrangement (season × production system). Season affected kikuyu chemical composition (*p* < 0.05) but not dry matter intake (DMI), milk production, quality, nor EMEs (*p* > 0.05). However, the absence of seasonal effects on these variables may be due to the sample size. Although HMY cows had a higher DMI (kg DM/d; *p* < 0.01) and EME (g/d, L/d; *p* < 0.05), they exhibited a lower methane intensity (both, L/L milk yield and L/kg fat-corrected milk) and gross energy intake lost as methane (*p* < 0.05). Positive correlations were found between EMEs and total dry matter intake (r = 0.638) and milk production (r = 0.726). The observed methane yield was comparable to previous studies for tropical kikuyu-based systems but lower than reports from temperate regions, suggesting seasonal-driven kikuyu quality does not translate into EME changes in high tropic regions. Animal productivity level was a key driver of EME magnitude and efficiency.

## Simple Summary

Enteric methane emissions from dairy cows represent an important source of greenhouse gases, yet they are poorly understood and rarely measured in tropical grazing systems. This study evaluated methane emissions in dairy cows grazing kikuyu grass in the Colombian high tropics using a sensor mask that measured exhaled gases directly in the field. Twelve cows with high or low milk production levels were monitored during low- and high-rainfall seasons. Although seasonal changes affected forage quality and composition, they did not significantly influence methane emissions, feed intake, or milk production. Instead, the cow’s productivity level played a more important role. Due to their greater intake, high-producing cows emitted more methane per day, but they were more efficient by emitting less methane per unit of milk and losing less energy as methane. Remarkably, methane emission per unit of dry matter intake was lower than previously reported values by the Intergovernmental panel on climate change (IPCC), suggesting that the IPCC system may overstate enteric emissions in tropical systems. The study also demonstrated that the sensor mask is a practical and affordable tool for measuring methane in grazing animals. These findings can help to improve the accuracy of greenhouse gas inventories and support more sustainable dairy production in tropical regions.

## 1. Introduction

The cattle population in Colombia ascends to 29.5 million heads [1], occupying 77.2% of the total agricultural area [2]. This activity generates more than 1.4 million direct jobs and represents over 21.8% of national agricultural gross domestic product [3] through beef and milk production, both of which are mainly pasture-based. Milk production in Colombia exceeded 8405 million liters [4] from various systems, among which specialized dairy operations contribute nearly 45% [5,6]. These systems are characterized by using high-yield dairy breeds (mostly Holstein and Jersey), introduced pastures such as kikuyu grass (*Cenchrus clandestinus*), and supplementation with starch-rich supplements [7]. They are located mainly in high-Andean regions and inter-Andean valleys in the Colombian tropics [8].

Kikuyu grass is the main forage in dairy operations in the high tropics of the Department of Antioquia, representing more than 70% of the grazing area in this region [9]. As a C4 grass, it accumulates higher amounts of structural carbohydrates (cellulose and hemicellulose), which reduce ruminal fermentability, DMI, and milk production [10]. Kikuyu’s chemical composition may vary throughout the year depending on seasonal variables such as ambient temperature, rain and solar radiation, affecting ruminal fermentability. Previous studies reported that kikuyu fermentability is higher during the high-precipitation season compared with the low-precipitation season [11]. To overcome these compositional limitations, producers have traditionally relied on supplementing commercial supplements rich in starch, which modify ruminal fermentation end products, including methane [12]. Methane (CH_4_) is a greenhouse gas with a higher warming potential than carbon dioxide and has received increased attention in recent years for estimating emissions from different production systems and identifying strategies for their mitigation [13]. Ruminal fermentation has been identified as one of the most important anthropogenic sources of CH_4_ [14].

The Intergovernmental panel on climate change (IPCC) has proposed several methodologies to estimate annual CH_4_ emissions based on assumptions that often do not cover the wide diversity of production conditions, potentially leading to inaccurate estimates. For Colombia, the IDEAM et al. uses the IPCC Tier 2 methodology for estimating enteric methane emissions (EMEs) from cattle [15] that involves the Agriculture, Forestry and Other Land Uses (AFOLU1 Colombia) model. The estimation of enteric fermentation, Tier 2 is based on models developed for animals under temperate production conditions, such as the NRC [16] beef cattle model and the Australian model for domesticated ruminants [17]. As a result, it is possible that the estimates do not fully correspond to the conditions under which these models were developed, generating errors and uncertainty. In addition, AFOLU 1 estimates EMEs by animal groups, creating noise when a specific animal type must be assigned to one of these categories, further increasing errors and uncertainty in the estimation process.

The most widely accepted methodology in temperate regions for measuring EMEs from grazing cattle involves CH_4_ quantification in continuous airflow collected during multiple visits to the feeding station of the GreenFeed System (C-lock Inc. Rapid City, SD, USA), which captures individual daily variation in CH_4_ emissions [18]. Despite the system estimating daily emissions using models based on repeated short-term visits, it cannot collect samples during periods of resting and rumination in grazing cattle [19]. To the best of our knowledge, studies on EME quantification under grazing conditions in Colombia are limited and have primarily relied on laser-based sensors to measure the CH_4_ concentration and estimate total emissions [20]. Therefore, reducing the uncertainty and improving the precision of estimates from cattle in countries such as Colombia is essential to properly quantify EMEs under grazing conditions. For this purpose, the electronic spirometry mask (ESM) has recently been proposed as a low-cost, easy-to-assemble device to which animals adapt quickly [21]. Jaimes et al. [21] reported that the ESM does not alter animal behavior nor change grazing capacity, covers only the nostrils, and is capable of measuring EMEs at a high temporal resolution during grazing activity. Therefore, the aim of this study was to quantify EMEs from dairy cows in the high tropical region of Antioquia during two seasons under two milk production management systems. We hypothesize that seasonal differences and milk production management may affect forage nutritional quality, influencing ruminal fermentation and, thereby, CH_4_ production.

## 2. Materials and Methods

### 2.1. Experimental Animals

This experiment was conducted at the Paysandú Agricultural Station of the Universidad Nacional de Colombia, located east of Medellín (Antioquia), at 2200 m above sea level, with a mean annual temperature of 16 °C and average relative humidity above 75%, characteristic of the bH–MB ecological zone [22]. All research procedures involving animals were reviewed and approved by the Institución Universitaria Vision de las Americas Ethic Committee (CICUA), protocol number 62 from 28 June 2023.

The flock was divided into two adjacent areas assigned to two management systems: one assigned to high-producing cows, managed with a more intensive fertilization plan and shorter pasture rest periods (high-milk-yield management system: HMYMS) (N 6°12′24.263″ O 75°30′4.744″), and another designated for medium-to-low-producing cows (low-milk-yield management system: LMYMS) (N 6°12′19.948″ O 75°30′20.642″). Each area had an independent milking parlor where cows were milked at 12 h intervals. A portable weather station (Watchdog 2700, Spectrum Technologies, Inc., Plainfield, IL, USA) was installed in the milking parlor for the high-producing cows to record precipitation, relative humidity, and ambient temperature.

### 2.2. Experimental Design

During a high-precipitation period (April 2025) and a medium-to-low-precipitation period (July 2025), 12 adult lactating Holstein cows were selected: six with milk production above 30 L/day in the HMYMS, and six with production below 15 L/day in the LMYMS, grazing different paddocks. The animals grazed kikuyu (*Cenchrus clandestinus*) pastures with 30 to 33 days of regrowth (Table 1) and received supplementation (0.25 lb/L after 10 L of milk yield) with a supplement based on maize and soybean meal at each of the two daily milkings (Table 2). The experimental phase for each period (high and low precipitation) lasted 12 days.

At the start of the first evaluation season (April 2025), the experimental cows had 4.3 ± 1.21 calvings, 134 ± 17.6 days in lactation, weighed 600 ± 82.0 kg, and had a body condition score (BCS) [23] of 3.08 ± 0.25. Initial milk production in this first season was 12.7 ± 0.25 L/d for LMYMS cows and 36.8 ± 2.46 L/d for HMYMS cows. At the start of the second evaluation season (July 2025), the experimental cows had 4.2 ± 0.75 calvings, 134 ± 20.1 days in lactation, a live weight of 551 ± 84.4 kg, and had a BCS of 3.11 ± 0.25. Initial milk production in this second season was 13.9 ± 3.84 L/day for LMYMS cows and 35.5 ± 7.56 L/day for HMYMS cows. Ten experimental cows were less than six months pregnant and all were in good health.

During the study, 15 g/cow of chromium oxide (Cr) was supplied at each milking as an external indicator to estimate fecal output. The indicator was mixed with supplement (1:1) and pelleted to ensure complete consumption. From day 10 to 12 of the experimental period, milk production was recorded daily (morning and afternoon), and milk and fecal samples were collected (daily at each milking). Pasture and concentrate supplement samples were also collected (days 1, 4, 7, 10, and 12). On the final day of the experimental period, cows were weighed again and BCS was reassessed.

Milk samples were analyzed for protein, fat, lactose, and total solids using infrared spectroscopy (ISO 9622:2013) [24]. Pasture and supplement samples were analyzed for crude protein (CP), neutral detergent fiber (NDF), acid detergent fiber (ADF), ash, chromium (Cr), and gross energy (GE) following AOAC (2016) [25] methods. In vitro dry matter digestibility was determined (IVDMD) using the pepsin–cellulase technique [26]. Indigestible dry matter in fecal samples was determined in vitro after 144 h of incubation and used as an internal marker [27]. Forage dry matter intake (DMIf) was estimated using the following equation [28]:

DMIf, = ([iDMfc] × (H/0.8) − [iDMs] × DMIs)/[iDMf]

where DMIf is DMI from forage expressed in kg of DM/cow/d; iDMfc is the indigestible dry matter determined in feces; H/0.8 indicates the external marker recovery percentage (80%) [27]; iDMs is the indigestible dry matter determined in the supplement; DMIs is the dry matter intake of supplement; and iDMf is the indigestible dry matter determined in the pasture.

### 2.3. Enteric Methane Emissions (EMEs)

The adaptation to the electronic spirometry masks (ESMs) involved fitting each cow with a polyester halter between days 3 and 6. Then, a dummy mask was attached to the halter between days 7 and 10 [29]. On days 10 and 12 of each experimental period, the cows wore the ESM from 08:00 to 18:00 h. To measure CH_4_ emissions from each cow, the ESM units were equipped with an electrochemical CH_4_ sensor (MQ4, Hanwei Electronics, Zhengzhou, China) and a flow sensor (DN-40, Louchen ZM, Shenzhen, China), connected to an ARDUINO UNO board (model ONE R3 MEGA328P, Si Tai&SH Hengtai Store, Hong Kong, China) programmed to record exhaled air volume (L/min) and CH_4_ concentration (ppm) in exhaled air every 500 ms onto a MicroSD memory card. The sensors were calibrated according to the procedure described by Jaimes et al. [21] before the start of each experimental period (Appendix A describes the detailed calibration of the MQ4 sensor). Raw data were recorded in Excel (Microsoft) for further analysis, and EME was calculated as follows:

CH_4_ = CH_4ppm_ × E × 60 × 24/1,000,000

where CH_4_ is expressed in L/cow/d; CH_4ppm_ is the methane concentration in exhaled air (ppm) and E is the volume of exhaled air (L/min).

### 2.4. Statistical Analysis

The effects of season and the milk production management systems (LMYMS or HMYMS) on pasture chemical composition were analyzed using a completely randomized design with a 2 × 2 factorial arrangement (season (S) × production level management systems (L)) using the PROC GLM procedure of SAS 9.4 (SAS Institute Inc, Cary, NC, USA). Statistical analyses of the performance variables measured in the animals were conducted under a completely randomized design with a 2 × 2 factorial arrangement (season × production level management systems) using the PROC MIXED procedure of SAS software (SAS Institute Inc., Cary, NC, USA) (1998) by applying the following mixed model:

Y_ijk_ = µ + L_i_ + S_j_ + LS_ij_ + C_j_(L)_i_ + ε_ijk_

where Y_ijk_ is the dependent variable observed for cow *k*, within production level *i*, during season *j*; μ = overall mean; L_i_ = fixed effect of production level management systems (i = low or high milk yield); S_j_ = fixed effect of season (j = rainy or dry season); (LS)_ij_ = fixed interaction effect between production level management systems and season; C_j_(L)_i_ = random effect of cow nested within production level management systems; and ε_ijk_ = residual error term. Mean comparisons were carried out with the LSMEANS test using the SAS statistical package. Differences were considered significant at *p* ≤ 0.05.

## 3. Results

Accumulated precipitation and relative humidity at the Paysandú Agricultural Station during April (high-precipitation season) were higher (356 mm and 87%, respectively) than those observed in July (medium–low-precipitation season) (222 mm and 83%, respectively). In contrast, average daily bright sunshine hours were higher during the medium–low-precipitation season (8.2 h/day) than during the high-precipitation season (6.5 h/day), whereas mean daily temperature was similar in both seasons (16.9 °C).

While no significant S × L interactions were detected for the chemical composition of kikuyu grass pastures (Table 1), some chemical fractions were affected by season (*p* < 0.05), except CP and in vitro dry matter digestibility (IVDMD), with no differences between pastures assigned to HMYMS and LMYMS cows (*p* > 0.05). The supplement offered to HMYMS cows had higher CP, GE, and IVDMD, but lower NDF, ADF, and ash content compared to the supplement fed to LMYMS cows (Table 2).

Season did not affect DMIf (*p* = 0.33), DMIs (*p* = 0.87), or total DMI (DMIt) (*p* = 0.40) and, consequently, did not affect gross energy intake (GEI) (Table 3). However, level of production affected all the intake parameters; thus, HMYMS cows showed higher DMIf, DMIs, DMIt, and GEI (*p* < 0.01). Similarly, season did not significantly affect variables associated with milk production and quality (*p* > 0.1; Table 4). In contrast, HMYMS cows exhibited a higher lactose content (*p* = 0.01) compared to LMYMS. A season × production level interaction was observed for milk fat content (*p* < 0.02), indicating that milk fat increased during the medium–low-precipitation season in HMYMS cows, whereas the opposite occurred in LMYMS cows.

Similarly, season did not affect the parameters associated with methane emissions (*p* > 0.1; Table 5). However, HMYMS cows showed a greater volume of exhaled air and higher total CH_4_ production, but lower CH_4_ emission intensity and a lower percentage of GE as CH_4_ (*p* < 0.04). The volume of methane emitted (L/d) showed an interaction effect. This is the result of the combined effect of the volume of exhaled air and its methane concentration. Of these factors, the volume of exhaled air was affected by the dairy production management system (*p* = 0.036). Therefore, since mathematically the methane concentration was higher in the LMYMS during the July season, its combination with the greater volume of exhaled air in this station resulted in a high volume of methane emission.

## 4. Discussion

### 4.1. Forage Chemical Composition

Kikuyu grass is a species with a rhizomatous and stoloniferous growth habit [30], which makes it more resilient to environmental changes. This is because both structures (rhizome, stolons) perform different but complementary functions in plants. Stolons act as supporting stems for photosynthesis, whereas rhizomes accumulate reserve carbohydrates and meristematic tissue, conferring a high degree of plasticity under varying environmental conditions [31]. These structures also ensure plant reproduction and expansion more efficiently than seeds [32], contributing to greater persistence over time. Despite these properties, in this experiment, the nutritional quality of kikuyu grass was affected by season, showing higher contents of CP, NDF, ADF, ash, and GE during the high-precipitation season than during the medium–low-precipitation season (Table 1). This may be explained by the fact that rainfall during the high-precipitation season was 60.4% greater and sunshine hours were 26.1% lower than in the medium-precipitation season, which could have affected soil nutrient uptake and the photosynthetic capacity of the pasture and, consequently, its chemical composition. Similarly, da Silva et al. [33] found that both precipitation and solar radiation influence the CP and NDF contents in several tropical grasses, including *Cynodon* spp. cultivar Tifton 85, another rhizomatous and stoloniferous species.

The chemical composition of the feed supplements offered to HMYMS and LMYMS cows during the two evaluation seasons falls within the values previously reported for this type of feed in northern Antioquia [10,34]. These supplements are formulated according to the animals’ milk yield and the average quality of the pastures offered, with the aim of improving the supply of energy and key nutritional fractions that are often limited in pastures, such as rumen-undegradable protein and non-structural carbohydrates [10].

### 4.2. Animal Performance

Differences in pasture chemical composition due to season did not affect pasture dry matter intake (DMIf; Table 3). However, it is necessary to take into account that this absence of seasonal effects on the DMIf may be due to the sample size and its possible impact on the statistical power. Similar results have been reported by other authors [35,36], which may be associated with the fact that DMIf in animals grazing high-NDF pastures is limited by rumen fill capacity [37,38]. Although some studies have reported a strong inverse relationship between NDF content and DMIf [39], others have shown high variability and low correlation between these two variables [40,41]. Thus, although in this experiment the difference in average pasture NDF content between seasons was 4.8%, it was apparently insufficient to promote a significant effect on rumen fill and, consequently, on DMIf.

Mertens [41] estimated that maximum NDF intake in lactating cows corresponded to 1.20% of body weight (BW). Later studies showed that this value was underestimated and could reach up to 1.83% of BW [42]. Data reported by Correa et al. [10] indicate that NDF intake from kikuyu grass may represent between 1.0 and 1.62% of the animals’ BW, suggesting considerable plasticity in animals’ capacity to respond to the effects of NDF on rumen fill. Likewise, Jaimes et al. [43] reported a similar maximum kikuyu NDF intake (1.57% of BW) with wide variability.

Another factor that may have contributed to the lack of a seasonal effect on forage dry matter intake (DMIf) is the substitution effect exerted by feed supplements, which also shows high variability, ranging from 0.11 to 0.50 kg supplement/kg forage [44]. In the current experiment, the average substitution rate (SR) was estimated at 0.336 ± 0.278 kg/kg. To determine this, the regression between supplement DMI (independent variable) and forage DMI (dependent variable) was first calculated (DMIf, kg/cow/d = 9.708 + 0.132 DMIs. r^2^ = 0.94). Then, the intercept (DMIf when cows are not supplemented) was incorporated into the formula proposed by Bargo et al. [45]:

SR (kg/kg) = (pasture DMI in the unsupplemented treatment (_kg/cow/d)_ − pasture DMI in the supplemented treatment (_kg/cow/d)_)/supplement DMI (_kg/cow/d)_.

As shown, the SR found in this study falls within the expected values but also shows high variability (CV = 82.9%), which may have contributed to the lack of seasonal effect on DMIf. Furthermore, because the milk production level was similar between the two assessed seasons and the concentrate supplementation was adjusted according to milk production, no differences in total DMI were observed between precipitation seasons (Table 3).

Pasture DMI (DMIf) was lower in LMYMS cows, likely due to their lower energy and nutrient requirements. The NRC [23], based on the theory of energy intake regulation, states that cows adjust intake according to their level of milk production. In this experiment, the correlation between kikuyu grass DMIf and milk yield was 0.748 (*p* < 0.01), slightly lower than the value reported by Correa et al. [10], which was 0.86 in Holstein cows grazing kikuyu pastures in Antioquia. Yet, because in dairy production systems in Antioquia the amount of supplement offered to lactating cows is adjusted to the milk production level, as was also done in this experiment, the correlation between supplement DMIs and milk yield was very high (0.993, *p* < 0.001). Overall, the correlation between DMIt and milk yield in this experiment was 0.983 (*p* < 0.001), notably greater than that reported by Bilal et al. [46] (r = 0.59), Britt et al. [47] (r = 0.44), and Madilindi et al. [48] (r = 0.32).

The lack of a seasonal effect on milk yield and quality (Table 4) is consistent with the absence of seasonal effects on both DMIf and DMIt. Although the production level did not affect body weight, milk from HMYMS, as expected, had lower fat and total solids contents but higher lactose (Table 4). These results agree with previous studies that reported a negative correlation between milk yield and milk fat [49,50], and between yield and total solids [50]. In contrast, Antanaitis et al. [51] and Barbosa et al. [49] reported a positive correlation between milk yield and lactose content.

### 4.3. Enteric Methane Emissions (EMEs)

The exhaled air volume of the experimental cows found in this study (Table 5) fell within the expected range for cows with the body weights reported here. According to Gallivan et al. [52], pulmonary ventilation in adult cattle averages 0.218 ± 0.044 L/kg/min, a range that includes the values observed in this study (0.232 ± 0.029 L/kg/min). Gallivan et al. [53] later reported that pulmonary ventilation in adult cows can reach 0.244 ± 0.035 L/kg/min, while Zhou et al. [54] indicated that Holstein cows under a 16 °C environment may exhibit a pulmonary ventilation rate of 145 L/min for cows weighing approximately 680 kg, equivalent to 0.216 L/kg/min. In this experiment, HMYMS cows showed a greater exhaled air volume (L/min), likely associated with increased metabolic activity and oxygen demand for milk production [55,56]. Similarly, Pinto et al. [57] reported an increased respiratory rate in higher-producing dairy cows, attributed to greater metabolic demands.

The methane concentrations in exhaled air observed in this study were higher than those reported by other authors who used different measurement techniques than the one employed in this experiment. These differences are due to the fact that the collected concentrations depend largely on airflow, dilution, sampling location, and equipment characteristics, among other factors. Thus, the methane concentrations shown in Table 5 were greater than those reported by Antanaitis et al. [58], who measured EMEs in housed Holstein cows using a laser detector and found concentrations ranging from 187 to 348 ppm. Likewise, Difford et al. [59] reported an average CH_4_ concentration of 410 ppm in Holstein and Jersey cows measured in respiration chambers, a value slightly higher than that reported by van Breukelen et al. [60] in the Netherlands using the GreenFeed system (average 325 ppm in cows with more than 75% Holstein genetics), and by Sahraei et al. [61], who reported an average of 235 ppm in lactating Holstein cows using the Sniffer system. In contrast, the CH_4_ concentrations reported in Table 5 fall within the range reported by Washburn and Brody [62] (1010 to 4190 ppm), who used spirometry masks that completely covered the muzzle of lactating cows.

Methane production (L/cow/day) (MP) is a raw metric influenced by various factors, particularly feed intake level and diet digestibility [63,64]. The MP values found in this study for HMYMS cows are higher than those reported by Noguera and Posada [65] (402 ± 40 L/cow/day), who measured MP using a respiration chamber in lactating Holstein cows consuming kikuyu grass. This difference may be explained by the lower milk production level of HMYMS cows (26.9 ± 1.9 L/cow/day) in the experimental cows of Noguera and Posada [65]. The MP values found here for LMYMS cows are similar to those reported by Molina et al. [66] in Holstein × Jersey crossbred cows grazing kikuyu grass, where EME was measured using the polytunnel method. However, these values are lower than those reported by Ulyatt et al. [67] (363 g/cow/day), who used the SF_6_ technique with lighter cows (438 kg body weight) grazing kikuyu grass in New Zealand, where the pasture NDF content was nearly 20% lower than in the present study. Likewise, the values for HMYMS cows found here are lower than those reported by Montenegro et al. [68] in Holstein cows in Costa Rica (390 g/cow/day) grazing a mixture of African star grass (*Cynodon nlemfuensis*) and kikuyu but receiving more than 14.0 kg/cow/day of feed supplements, likely with higher digestibility than that observed here (Table 1 and Table 2).

Because no differences were found in forage intake, supplement intake, or DMIt between evaluation periods (Table 3), no effect of season on CH_4_ production was observed (Table 5). Nevertheless, MP was higher in HMYMS cows (*p* < 0.016), reflecting their greater pasture, supplement, and DMIt. Accordingly, the correlation between milk yield level and MP was high and positive (r = 0.726. *p* < 0.007), as was the correlation between total DMI and MP (r = 0.638. *p* < 0.027). Similar relationships have been reported by other authors [69,70,71] and have been used to develop regression equations for estimating CH_4_ production [72,73].

Methane yield (g/kg DMIt) (MY) is a more refined EME metric that reflects the efficiency with which consumed feed is converted into CH_4_. In this experiment, MY was similar to that reported by Noguera and Posada [65] (16.1 ± 4.0 g/kg DMI) in Holstein cows consuming kikuyu grass, but higher than that reported by Molina et al. [66] in Holstein × Jersey crossbred cows grazing kikuyu grass, likely due to their lower reported DMI. In contrast, Montenegro et al. [68] reported higher MY values in Holstein cows grazing African star grass (*Cynodon nlemfuensis*) and kikuyu pastures in Costa Rica (20.5 ± 1.3 g/kg DMI), despite similar milk yield and DMI levels to those exhibited by HMYMS cows in this study; this difference may be explained by their higher MP values. The MY factors currently used in other regions of the world are generally higher than those observed here. For example, average values of 18.2 ± 3.71 and 21.4 ± 3.39 g CH_4_/kg DMI have been reported in the United States and Europe, respectively [74], while in New Zealand, the value is 22.1 g/kg DMI [64]. Methane intensity (L CH_4/_FCM or g CH_4/_kg milk yield) (MI) is perhaps the most relevant efficiency metric for evaluating the environmental impact of dairy systems [75] and has long been suggested to be included in dairy genetic selection programs to improve efficiency traits [76].

Measurements of methane emitted by ruminants have been published since the 19th century [77,78], when this gas was simply called “hydrocarbon” (Kohlenwasserstoff) or “swamp gas” (sumpgas), and its chemical formula was considered to be CH_2_. Although the caloric values of methane were known from 1838 [79], the idea that methane excreted by animals represents an energy loss was not introduced until the beginning of the 20th century [80]. It was incorporated into energy systems in the calculus of metabolizable energy by Armsby [81]. The percentage of gross energy intake lost as CH_4_ (Ym) [82] has been referred to by other authors as the “methane conversion factor” [83], “methane emission factor” [84], or “cattle methane yield” [85]. This parameter was particularly important in energy studies in ruminants until the 1970s [63]. However, when CH_4_ was classified as a greenhouse gas in the mid-1970s [86], its importance in studies on ruminant nutrition, physiology, and metabolism increased significantly, and today, its relevance as a source of energy loss in ruminants is reinforced by its importance as a greenhouse gas. The Ym showed in Table 5 are between the range reported by Moe and Tyrrell [63] (1.6 to 9.9%) in their analysis of energy balance trials with Holstein cows, and in the range reported by Niu et al. [74] in an intercontinental database (2.7 to 9.8%), but are lower than the Ym proposed by the IPCC [87] for dairy cows (5.7 to 6.5%), indicating that the values of the IPCC may overestimate methane emission, as previously indicated by Noguera and Posada [65]. These authors reported a mean Ym of 4.9 ± 1.2% in Holstein cows under a respirometry chamber in Northern Antioquia with a mean milk yield of 26.9 ± 1.9 L/d.

In this experiment, while Ym was positively correlated with MY (r = 0.606. *p* < 0.036) and with MI (r = 0.786. *p* < 0.003), it was not correlated with MP (r = 0.047. *p* > 0.885), suggesting that the last metric may not be an appropriate indicator for the analysis of methane emission in ruminants under the evaluated experimental conditions. However, Ym is affected by several factors including dry matter intake, feed nutrient composition [74,83], genetic background [88], and milk yield [89]. In this experiment, Ym was correlated negatively with total DMI (r = −0.728. *p* < 0.0074) and fat-corrected milk yield (r = −0.639. *p* < 0.025), indicating that with a higher intake and higher milk fat-corrected yield, a lower gross energy intake is lost in the form of methane, as was observed by others [71,88].

The use of a spirometry mask represents a valuable alternative for quantifying EMEs because it enables direct and continuous measurement of exhaled gases during behavioral states including grazing, rumination, and resting, which are typically underrepresented or not directly determined by other techniques (i.e., GreenFeed system [90,91], SF_6_ [92], respiration chamber [93,94]). The spirometry mask allows for high-frequency, real-time measurements of the CH4 concentration and airflow to characterize emissions during rumination and idling periods when CH_4_ production remains substantial but is often unmeasured by other systems. This capacity to capture temporal variability improves the accuracy of daily EME estimates and may provide a more mechanistic understanding of emission dynamics in grazing cattle systems. Finally, it is important to highlight that, based on continuous observation of the animals wearing the ESM, they did not exhibit any behaviors indicating discomfort. This type of response has been previously reported with this type of mask [21] and even with masks that completely cover the snout [95,96,97], suggesting that they have a minimal effect on their food and water intake.

## 5. Conclusions

Under the conditions of this study, DMI and EME were primarily driven by production level rather than rainy season, as no seasonal effects were observed on intake, milk yield or CH_4_ production. However, the absence of seasonal effects on these variables may be due to the sample size. HMYMS cows in the evaluated environment exhibited greater pasture, supplement and total DMI, which translated into greater absolute EME, while CH_4_ yield remained within the reported ranges for grazing systems based on kikuyu grass. Strong positive correlations between milk yield, total DMI and CH_4_ production confirm that intake is a major determinant of EME output. Despite differences in milk composition associated with production level, overall animal performance and CH_4_ intensity reflected biological relationships, supporting the robustness of intake-driven prediction of EMEs in grazing systems. The observed exhaled air volumes and CH_4_ concentrations were consistent with the literature values, indicating that the spirometry mask methodology provides feasible field estimates of respiratory exchange and CH_4_ output under grazing conditions. The values of Ym found in this experiment agreed with other reports but were lower than the values proposed by the IPCC.

## Figures and Tables

**Table 1:** Chemical composition of kikuyu grass (*Cenchrus clandestinus*) pastures assigned to experimental cows in the high- and low-milk-yield management systems during the two evaluation seasons.

	Management System ^2^							
Item ^1^	HMYMS		LMYMS			*p*-Value ^6^		
% of DM	High ^3^	Med ^4^	High ^3^	Med ^4^	SEM ^5^	S	L	S × L
CP	20.0	17.8	18.1	17.8	0.96	0.053	0.132	0.121
NDF	67.1	62.2	67.9	63.1	3.57	0.002	0.467	0.953
ADF	37.8	35.0	37.5	34.8	3.77	0.040	0.807	0.966
Ash	8.58	7.11	8.98	7.75	0.37	0.005	0.179	0.733
IVDMD, %	49.8	46.7	48.3	47.9	2.71	0.214	0.513	0.380
GE, Mcal/kg	4.38	4.10	4.34	4.12	0.01	0.006	0.864	0.680

^1^ CP: crude protein; NDF: neutral detergent fiber; ADF: acid detergent fiber; IVDMD: in vitro dry matter digestibility; GE: gross energy. ^2^ HMYMS: high-milk-yield management system cows; LMYMS: low-milk-yield management system cows. ^3^ High: season of high precipitation. ^4^ Med: season of medium–low precipitation. ^5^ Pooled standard error of treatment means, n = 6 cows. ^6^ Observed significance level for the effect of season (S), level of milk yield management system (L), and their interaction (S × L).

**Table 2:** Chemical composition of the feed supplements offered to high- and low-milk-yield management system cows during the evaluated seasons.

	Management System ^2^			
Item ^1^	HMYMS		LMYMS	
% of DM	High ^3^	Med ^4^	High ^3^	Med ^4^
CP	15.60	16.10	11.30	13.30
NDF	21.80	20.90	40.40	43.40
ADF	12.80	12.30	25.30	27.20
Ash	7.87	8.22	11.60	9.75
IVDMD, %	80.70	83.50	61.01	57.50
GE, Mcal/kg	4.26	4.24	4.07	4.17

^1^ CP: crude protein; NDF: neutral detergent fiber; ADF: acid detergent fiber; IVDMD: in vitro dry matter digestibility; GE: gross energy. ^2^ HMYMS: high-milk-yield management system cows; LMYMS: low-milk-yield management system cows. ^3^ High: season of high precipitation. ^4^ Med: season of medium–low precipitation.

**Table 3:** Forage, supplement and total intake of experimental cows in high- and low-milk-yield management system cows during the two evaluation periods.

	Management System ^2^								
Item ^1^	HMYMS		LMYMS				*p*-Value ^7^		
kg/cow/d	High ^3^	Med ^4^	High ^3^	Med ^4^	SEM ^5^	CI ^6^	S	L	S × L
DMIf	11.8	12.9	10.6	10.5	0.52	11.4 ± 0.7	0.339	0.01	0.319
DMIs	8.3	7.5	2.4	3.3	0.37	5.4 ± 1.5	0.864	0.001	0.058
DMIt	20.0	20.4	13.0	13.9	0.69	16.8 ± 2.1	0.403	0.001	0.696
DMD. %	72.3	71.3	65.8	66.5	1.27	69.0 ± 1.9	0.898	0.002	0.513
GEI, Mcal/d	87.5	87.0	54.6	58.8	2.97	71.6 ± 9.1	0.412	0.001	0.673

^1^ DMIf: dry matter intake of forage; DMIs: dry matter intake of supplement; DMIt: total dry matter intake; DMD: dry matter digestibility; GEI: gross energy intake. ^2^ HMYMS: high-milk-yield management system cows; LMYMS: low-milk-yield management system cows. ^3^ High: season of high precipitation. ^4^ Med: season of medium–low precipitation. ^5^ Pooled standard error of treatment means. n = 6 cows. ^6^ CI: confidence interval. ^7^ Observed significance level for the effect of season (S), level of milk yield management system (L), and their interaction (S × L).

**Table 4:** Body weight, milk yield, and milk quality of experimental cows in the high- and low-milk-yield management systems during the two evaluation periods.

	Management System ^2^								
Item ^1^	HMYMS		LMYMS				*p*-Value ^7^		
	High ^3^	Med ^4^	High ^3^	Med ^4^	SEM ^5^	CI ^6^	S	L	S × L
BW. kg	594	616	607	487	43.0	576 ± 47	0.281	0.214	0.137
Yield. L/cow/d	37.5	35.6	13.0	17.8	1.88	26.0 ± 6.5	0.463	0.001	0.110
Fat. %	2.53	2.93	4.07	3.59	0.14	3.3 ± 0.37	0.786	0.001	0.015
CP. %	3.3	3.15	3.72	3.21	0.14	3.3 ± 0.17	0.051	0.135	0.250
Lactose. %	5.2	4.92	4.27	4.57	0.19	4.7 ± 0.26	0.954	0.011	0.179
Solids. %	12.0	11.9	12.9	12.3	0.42	12.3 ± 0.42	0.408	0.171	0.518
FCM. L/cow/d	29.3	29.9	13.1	16.8	1.77	18.8 ± 3.1	0.264	0.001	0.423

^1^ BW: body weight; Yield: individual milk yield; CP: milk crude protein; Solids: total solids in milk; FCM: 4% fat-corrected milk. ^2^ HMYMS: high-milk-yield management system cows; LMYMS: low-milk-yield management system cows. ^3^ High: season of high precipitation. ^4^ Med: season of medium–low precipitation. ^5^ Pooled standard error of treatment means. n = 6 cows. ^6^ CI: confidence interval. ^7^ Observed significance level for the effect of season (S), level of milk yield management system (L), and their interaction (S × L).

**Table 5:** Exhaled air volume and methane emissions of high- and low-milk-yield management system cows during the evaluation seasons using the spirometry mask method.

	Management System ^2^								
Item ^1^	HMYMS		LMYMS				*p*-Value ^7^		
	High ^3^	Med ^4^	High ^3^	Med ^4^	SEM ^5^	CI ^6^	S	L	S × L
Exhal. L/min	149	135	111	127	9.10	130 ± 11	0.904	0.036	0.142
Methane									
Concentration. ppm	1975	2084	1937	2125	78.9	2030 ± 80	0.096	0.989	0.628
Volume. L/d	423	406	303	388	19.9	380 ± 32	0.128	0.009	0.032
Volume. g/d	303	290	217	278	14.2	272 ± 23	0.128	0.001	0.032
Yield. L/kg DMIt	21.2	24.6	23.5	24.6	2.05	23.5 ± 1.5	0.166	0.451	0.451
Yield. g/kg DMIt	15.2	17.6	16.8	17.6	1.05	16.8 ± 1.1	0.174	0.467	0.467
Intensity. L/L milk	11.3	11.6	23.5	22.1	1.70	17.1 ± 3.6	0.739	0.001	0.627
Intensity. L/FCM	14.5	13.8	23.2	23.6	1.73	18.8 ± 3.1	0.926	0.001	0.766
Ym. %	4.62	4.36	5.18	6.16	0.36	5.1 ± 0.5	0.346	0.012	0.124

^1^ Exhal: exhalations. Ym = percentage of gross energy intake lost as CH_4_ (% of gross energy intake). ^2^ HMYMS: high-milk-yield management system cows; LMYMS: low-milk-yield management system cows. ^3^ High: season of high precipitation. ^4^ Med: season of medium–low precipitation. ^5^ Pooled standard error of treatment means. n = 6 cows. ^6^ CI: confidence interval. ^7^ Observed significance level for the effect of season (S), level of milk yield management system (L), and their interaction (S × L).

## Data Availability

Data are available upon request to the corresponding author.

## Abbreviations

The following abbreviations are used in this manuscript:

EME	Enteric methane emissions
ESM	Electronic spirometry mask
HMYMS	High-milk-yield management system
LMYMS	Low-milk-yield management system
DMI	Dry matter intake
CH_4_	Methane
BW	Body weight
BCS	Body condition score
CP	Crude protein
NDF	Neutral detergent fiber
ADF	Acid detergent fiber
Cr	Chromium
GE	Gross energy
iDM	Indigestible dry matter
IVDMD	In vitro dry matter digestibility
FCM	4% fat-corrected milk
Ym	Percentage of gross energy intake lost as CH_4_

## Appendix A

MQ4 is a specific methane sensor with a lifespan of nearly 10 years. It is composed of a micro AL2O_3_ ceramic tube, tin dioxide (SnO_2_) sensitive layer, the measuring electrode and the heater. At operating temperature, atmospheric oxygen chemisorbs onto the SnO_2_ surface, capturing electrons and increasing the material’s electrical resistance. When methane molecules arrive, they react with the chemisorbed oxygen, releasing electrons back into the SnO_2_ and reducing its resistance. The higher the methane concentration, the lower the sensor resistance (Rs) relative to its baseline resistance in clean air (R0) (MQ4 Technical Data, Hanwei Electronics, Zhengzhou, China).

Before starting the calibration of the MQ4 sensors, they were subjected to a preheating process for 24 h by connecting them to a 5v source.

**Calibration of MQ4 sensor**

**Internal calibration**

**Step 1:**

Obtain each data point from the MQ4 data sheet for both CH_4_ and clean air use, for example, WebPlotDigiter 4.7. Then, calculate the natural logarithm of each data point and estimate the linear regression equation for clean air. The slope and intercept values will be used in step 4. In this experiment, we calculated these values to be 1.4861 and −0.0016, respectively.

**Step 2: Calculation of R0**

After the preheating step, the MQ4 is connected to the ARDUINO UNO board, which in turn is connected to a USB port on the computer and programmed using the ARDUINO IDE software, version 2:

void setup() {

 Serial.begin(9600); // Set baud rate

}

void loop() {

 float sensor_volt;

 float RS_air;   

 float R0;     

 float sensorValue = 0;

 // Take 500 samples for averaging

 for (int x = 0; x < 500; x++) {

  sensorValue += analogRead(33); // Sum analog values of the sensor

 }

 sensorValue = sensorValue / 500.0;

 // Convert the average sensor value to voltage

 sensor_volt = sensorValue * (4.95 / 4095.0);

 // Calculate sensor resistance in fresh air (RS_air)

 RS_air = ((4.95 * 10.0) / sensor_volt) - 10.0;

 // Calculate R0 (baseline resistance in clean air)

 R0 = RS_air / 4.3;

 // Print the R0 value to the serial monitor

 Serial.print("R0 = ");

 Serial.println(R0);

 delay(1000);

}

**Step 3: Calculation of Rs/R0 ratio**

void setup() {

 Serial.begin(9600);

}

void loop() {

  float sensor_volt;

 float RS_gas;

 float ratio;

 //-Replace the name "R0" with the value of R0 in the demo of First Test -/

 float R0 = 0.91;

 int sensorValue = analogRead(A0);

 sensor_volt = ((float)sensorValue / 1024) * 5.0;

 RS_gas = (5.0 - sensor_volt) / sensor_volt; // Depend on RL on yor module

 ratio = RS_gas / 55; // ratio = RS/R0

  Serial.print("sensor_volt = ");

 Serial.println(sensor_volt);

 Serial.print("RS_ratio = ");

 Serial.println(RS_gas);

 Serial.print("Rs/R0 = ");

 Serial.println(ratio);

 Serial.print("\n\n");

 delay(1000);

}

**Step 4: Calculation of CH_4_ concentration in ppm**

#define gas_sensor 0 // Sensor pin (ESP32 GPIO pin)

float m = -0.0016;  // Slope (verify with MQ-4 datasheet)

float b = 1.4861;   // Y-Intercep t (verify with MQ-4 datasheet)

float R0 = 56;   // Calibrated sensor resistance in clean air

void setup() {

 Serial.begin(9600); // Start serial communication

}

void loop() {

 float sensor_volt;

 float RS_gas;

 float ratio;

 float sensorValue;

 // Read sensor value from analog pin

 sensorValue = analogRead(gas_sensor);

 // Convert sensor value to voltage (using 4.99V supply)

 sensor_volt = sensorValue * (4.95 / 4095.0);

 // Calculate RS_gas (sensor resistance in the current environment)

 RS_gas = ((4.95 * 10.0) / sensor_volt) - 10.0;

 // Calculate the ratio of RS_gas to R0

 ratio = 0.19;

 // Calculate gas concentration in ppm (logarithmic scale)

 double ppm_log = (log10(ratio) - b) / m;

 double ppm = pow(10, ppm_log);

 // Display ppm value on the Serial Monitor

 //Serial.print("Gas Concentration (ppm): ");

 //Serial.println(ppm);

 Serial.print("Gas Concentration (analog read): ");

 Serial.println(analogRead(0));

 delay(1000);

}

**Step 5: Establish CH_4_ concentration in zero**

After the methane values stabilize, use this value in the code “Serial.println(analogRead(0)+ or - value of Step 3);” to obtain the value of zero by adding or subtracting as appropriate.

**External calibration**

**Step 6:**

Once the internal calibration was completed, the MQ4 sensors were calibrated with six concentrations of methane gas (0, 500, 1000, 1500, 2000, 2500, and 3000 ppm) obtained from a standard cylinder containing 1.45% methane (14,500 ppm) (Reference 10103262, MSA The Safety Company, Cranberry, Cranberry Township, PA, USA) and diluted in a 2 L bottle with CO_2_. The concentration values obtained with the MQ4 sensors were compared with those obtained using a methane analyzer with an N55A 22YS catalytic combustion sensor (Nemoto Special Chemicals Co., Ltd., Tokyo, Japan) calibrated in a certified laboratory (MLS Laboratory, Medellín, Colombia). Then, a linear regression equation was calculated to estimate the methane concentration from the data obtained from the MQ4 sensor and included in the final reading code:

void setup()

{

 Serial.begin(9600); //Set serial baud rate at 9600 bps

}

void loop()

{

int sensorValue = analogRead(A0);

  // print out the value you read:

  Serial.print("CH4: ");

  Serial.println((sensorValue*slope)+ or - intercep);// The values are an example after calibration

  delay(100);

}

Now, since cow exhalations are permanently warm and humid, it was not necessary to compensate for methane measurements associated with variations caused by these variables [98].

## References

[1] Federación Nacional de Ganaderos (FEDEGAN) (2025). Balance y Perspectivas del Sector Ganadero Colombiano 2024–2025.

[2] Departamento Administrativo Nacional de Estadística (DANE) (2023). Encuesta Nacional Agropecuaria 2023.

[3] Unidad de Planificación Rural Agropecuaria (UPRA) (2025). Sistema Productivo en Ganadería Bovina Doble Propósito.

[4] Federación Colombiana de Ganaderos (FEDEGAN) (2026). Industry Figures: Milk Production and Collection in Colombia (Liters).

[5] Departamento Administrativo Nacional de Estadística (DANE) (2017). Boletín Técnico. Encuesta Nacional Agropecuaria 2017.

[6] Unidad de Planificación Rural Agropecuaria (UPRA) (2020). Plan de Ordenamiento Productivo para la Cadena Láctea Bovina en Colombia.

[7] Carulla J.E., Ortega E. (2016). Sistemas de producción lechera en Colombia: Retos y oportunidades. Arch. Latinoam. Prod. Anim..

[8] Morales-Vallecilla F., Ortiz-Grisales S. (2018). Productividad y eficiencia de ganaderías lecheras especializadas en el Valle del Cauca (Colombia). Rev. Fac. Med. Vet. Zootec..

[9] Jaimes Cruz L.J., Correa Cardona H.J. (2016). Balance de nitrógeno, fósforo y potasio en vacas Holstein pastando praderas de kikuyo (*Cenchrus clandestinus*) en el norte de Antioquia. CES Med. Vet. Zootec..

[10] Correa H.J., Pabón M.L., Carulla J.E. (2008). Valor nutricional del pasto kikuyo (*Pennisetum clandestinum* Hoechst Ex Chiov.) para la producción de leche en Colombia (Una revisión): I—Composición química y digestibilidad ruminal y posruminal. Livest. Res. Rural Dev..

[11] Correa H.J., Escalante L.F., Jaimes L.J. (2018). Efecto de la época del año y la altura remanente posterior al pastoreo sobre el crecimiento y calidad nutricional del pasto kikuyo (*Cenchrus clandestinus*) en el norte de Antioquia. Livest. Res. Rural Dev..

[12] Ramírez J., Posada S., Noguera R. (2015). Effects of Kikuyu grass (*Pennisetum clandestinum*) age and different forage: Concentrate ratios on methanogenesis. Rev. MVZ Córdoba.

[13] Arndt C., Hristov A.N., Price W.J., McClelland S.C., Pelaez A.M., Cueva S.F., Oh J., Dijkstra J., Bannink A., Bayat A.R. (2022). Full adoption of the most effective strategies to mitigate methane emissions by ruminants can help meet the 1.5 °C target by 2030 but not 2050. Proc. Natl. Acad. Sci. USA.

[14] Shibata M., Terada F. (2010). Factors affecting methane production and mitigation in ruminants. Anim. Sci. J..

[15] IDEAM., Fundación Natura, PNUD., MADS., DNP., Cancillería (2021). Tercer Informe Bienal de Actualización de Colombia a la Convención Marco de las Naciones Unidas para el Cambio Climático (CMNUCC).

[16] National Research Council (NRC) (2000). Nutrient Requirements of Beef Cattle.

[17] Commonwealth Scientific and Industrial Research Organization (CSIRO) (2007). Nutrient Requirements of Domesticated Ruminants.

[18] van Gastelen S., Dijkstra J., Heck J.M.L., Kindermann M., Klop A., de Mol R., Rijnders D., Walker N., Bannink A. (2022). Methane mitigation potential of 3-nitrooxypropanol in lactating cows is influenced by basal diet composition. J. Dairy Sci..

[19] Garnett E. (2012). Evaluation of the GreenFeed System for Methane Estimation from Grazing Dairy Cows. Master’s Thesis.

[20] Narváez-Herrera J.P., Angulo-Arizala J., Barragán-Hernández W.A., Mahecha-Ledesma L. (2026). Estimation of enteric methane emissions in dairy cows under grazing a silvopastoral system and a grass monoculture in the Colombian Amazonian foothills. PLoS ONE.

[21] Jaimes L.J., Castrillón S., Bustamante B.S., Correa H.J. (2025). Through the mouth or nostrils: The methane excretion route in belching dairy cows. Animals.

[22] Espinal T.L.S. (1990). Zonas de Vida de Colombia.

[23] National Research Council (NRC) (2021). Nutrient Requirements of Dairy Cattle.

[24] (2013). Milk and Liquid Milk Products—Guidelines for the Application of Mid-Infrared Spectrometry.

[25] AOAC (2016). Official Methods of Analysis of the Association of Official Analytical Chemists.

[26] Navarro-Ortiz C.A., Roa-Vega M.L. (2018). Comparación de la digestibilidad de tres especies forrajeras estimada mediante diferentes técnicas. Orinoquia.

[27] Correa H.J., Pabón M.L., Carulla J.E. (2009). Estimación del consumo de materia seca en vacas Holstein bajo pastoreo en el trópico alto de Antioquia. Livest. Res. Rural Dev..

[28] Geerken C.M., Calzadilla D., González R. (1987). Aplicación de técnica de dos marcadores para medir el consumo de pasto y la digestibilidad de la ración de vacas en pastoreo suplementadas con concentrado. Pastos Forrajes.

[29] Correa Cardona H.J., Jaimes Cruz L.J. (2023). Design and operation of a spirometry mask to quantify exhaled methane emission by grazing cattle. Livest. Res. Rural Dev..

[30] Davidse G., Sousa M.S., Chater A.O. (1994). Flora Mesoamericana, Vol. 6: Alismataceae a Cyperaceae.

[31] Dong M., Pierdominici M.G. (1995). Morphology and growth of stolons and rhizomes in three clonal grasses, as affected by different light supply. Vegetatio.

[32] Guo L., Plunkert M., Luo X., Liu Z. (2021). Developmental regulation of stolon and rhizome. Curr. Opin. Plant Biol..

[33] da Silva E.A., Silva W.J., Barreto A.C., Oliveira A.B., Paes J.M.V., Ruas J.R.M., Queiroz D.S. (2012). Chemical composition and photosynthetically active radiation of forage grasses under irrigation. Rev. Bras. Zootec..

[34] González C., Correa H.J. (2007). Factores nutricionales y alimenticios que afectan la producción de leche y el contenido de proteína en la leche en hatos especializados de Antioquia. Despertar Leche..

[35] Mudavadi O.P., Mpolya A.E., Gachuiri C., Muyekho F.N., Lukuyu A.B. (2020). Effects of season variation on water, feed, milk yield and reproductive performance of dairy cows in smallholder farms in Eastern Africa. J. Agric. Ecol. Res. Int..

[36] Burgers E.E.A., Koning L., Pellikaan W., Holshof G., Klop A., Kar-Klootwijk C.C.W. (2025). Comparing individual grass intake of dairy cows in three grass-based systems using three methods: Automated weighing bins, net energy evaluation, and *n*-alkanes. Grass Forage Sci..

[37] Decruyenaere V., Buldgen A., Stilmant D. (2009). Factors affecting intake by grazing ruminants and related quantification methods: A review. Biotechnol. Agron. Soc. Environ..

[38] Mertens D.R. (2009). Impact of NDF content and digestibility on dairy cow performance. Adv. Dairy Technol..

[39] Arelovich H.M., Abney C.S., Vizcarra J.A., Galyean M.L. (2008). Effects of dietary neutral detergent fiber on intakes of dry matter and net energy by dairy and beef cattle: Analysis of published data. Prof. Anim. Sci..

[40] Allen M.S. (2000). Effects of diet on short-term regulation of feed intake by lactating dairy cattle. J. Dairy Sci..

[41] Mertens D.R. (1985). Factors influencing feed intake in lactating cows: From theory to application using neutral detergent fiber. Proceedings of the Georgia Nutrition Conference.

[42] Bargo F., Muller L.D., Delahoy J.E., Cassidy T.W. (2002). Milk response to concentrate supplementation of high producing dairy cows grazing at two pasture allowances. J. Dairy Sci..

[43] Jaimes L.J., Cerón J.M., Correa H.J. (2015). Season and stage of lactation affects feed intake of Holstein cows grazing Kikuyo (*Cenchrus clandestinus*) in Colombia. Livest. Res. Rural Dev..

[44] Mayne C.S., Wright I.A., Garnsworthy P.C. (1988). Herbage intake and utilization by the grazing dairy cow. Nutrition and Lactation in the Dairy Cow.

[45] Bargo F., Muller L.D., Kolver E.S., Delahoy J.E. (2003). Invited review: Production and digestion of supplemented dairy cows on pasture. J. Dairy Sci..

[46] Bilal R.I., Cue R.I., Hayes J.F. (2016). Genetic and phenotypic associations of type traits and body condition score with dry matter intake, milk yield, and number of breedings in first-lactation Canadian Holstein cows. Can. J. Anim. Sci..

[47] Britt J.S., Thomas R.C., Speer N.C., Hall M.B. (2003). Efficiency of converting nutrient dry matter to milk in Holstein herds. J. Dairy Sci..

[48] Madilindi M.A., Banga C.B., Zishiri O.T. (2022). Prediction of dry matter intake and gross feed efficiency using milk production and live weight in first-parity Holstein cows. Trop. Anim. Health Prod..

[49] Boujenane I. (2002). Estimates of genetic and phenotypic parameters for milk production in Moroccan Holstein-Friesian cows. Rev. Elev. Med. Vet. Pays Trop..

[50] Barbosa S.B.P., Modesto E.C., Lopes F.A., da Silva E.C., Acosta A.C. (2020). Relationship between milk production system and milk traits and somatic cell counts in Brazilian Murrah buffaloes: A multivariate analysis. Acta Sci. Anim. Sci..

[51] Antanaitis R., Džermeikaitė K., Krištolaitytė J., Girdauskaitė A., Arlauskaitė S., Tolkačiovaitė K., Baumgartner W. (2024). The relation between milk lactose concentration and the rumination, feeding, and locomotion behavior of early-lactation dairy cows. Animals.

[52] Gallivan G.J., McDonell W.N., Forrest J.B. (1989). Comparative pulmonary mechanics in the horse and the cow. Res. Vet. Sci..

[53] Gallivan G.J., Viel L., Baird J.D., McDonell W.N. (1991). Pulmonary structure and function in adult dairy cows with an expanded lung field. Can. J. Vet. Res..

[54] Zhou M., Huynh T.T.T., Groot Koerkamp P.W.G., van Dixhoorn I.D.E., Amon T., Aarnink A.J.A. (2022). Effects of increasing air temperature on skin and respiration heat loss from dairy cows at different relative humidity and air velocity levels. J. Dairy Sci..

[55] Delamaire E., Guinard-Flament J. (2004). Adaptation of the mammary oxygen consumption in response to milking frequency variations in dairy cows. J. Anim. Feed Sci..

[56] West J.W. (2003). Effects of heat stress on production in dairy cattle. J. Dairy Sci..

[57] Pinto S., Hoffmann G., Ammon C., Heuwieser W., Levit H., Halachmi I., Amon T. (2019). Effect of two cooling frequencies on respiration rate in lactating dairy cows under hot and humid climate conditions. Ann. Anim. Sci..

[58] Antanaitis R., Anskienė L., Rapaliutė E., Bilskis R., Džermeikaitė K., Bačėninaitė D., Juškienė V., Juška R., Meškinytė E. (2022). Relationship between reticulorumen parameters measured in real time and methane emission and heat stress risk in dairy cows. Animals.

[59] Difford D.W., Olijhoek A.L.F., Hellwing A.L.F., Lund P., Bjerring M.A., de Haas Y., Lassen J., Løvendahl P. (2019). Ranking cows’ methane emissions under commercial conditions with sniffers versus respiration chambers. Acta Agric. Scand. A Anim. Sci..

[60] van Breukelen A.E., Aldridge M.N., Veerkamp R.F., Koning L., Sebek L.B., de Haas Y. (2023). Heritability and genetic correlations between enteric methane production and concentration recorded by GreenFeed and sniffers on dairy cows. J. Dairy Sci..

[61] Sahraei A., Knob D., Lambertz C., Gattinger A., Breuer L. (2025). Modeling enteric methane emission from dairy cows using deep learning approach. Sci. Total Environ..

[62] Washburn L.E., Brody S. (1937). Growth and development with special reference to domestic animals. XLII. CH4, hydrogen, and carbon dioxide production in the digestive tract of ruminants in relation to the respiratory exchange. Mo. Agric. Exp. Stn. Res. Bull..

[63] Moe P.W., Tyrrell H.F. (1979). Methane production in dairy cows. J. Dairy Sci..

[64] Muetzel S., Hannaford R., Jonker A. (2024). Effect of Animal and Diet Parameters on Methane Emissions for Pasture-Fed Cattle. Anim. Prod. Sci..

[65] Noguera R.R., Posada S.L. (2017). Factor de emisión de metano entérico para vacas Holstein lactantes en la zona norte de Antioquia-Colombia. Livest. Res. Rural Dev..

[66] Molina-Botero I.C., Gaviria-Uribe X., Rios-Betancur J.P., Medina-Campuzano M., Toro-Trujillo M., González-Quintero R., Ospina B., Arango J. (2024). Methane Emission, Carbon Footprint and Productivity of Specialized Dairy Cows Supplemented with Bitter Cassava (*Manihot esculenta* Crantz). Animals.

[67] Ulyatt M.J., Lassey K.R., Shelton I.D., Walker C.F. (2002). Methane emission from dairy cows and wether sheep fed subtropical grass-dominant pastures in midsummer in New Zealand. N. Z. J. Agric. Res..

[68] Montenegro-Ballestero J., Barrantes-Guevara E., Ivankovich-Cruz S. (2020). Cuantificación de metano entérico según estado fisiológico en vacas lecheras de alta producción en Costa Rica. Agron. Costarric..

[69] Kennedy M., Lahart B., Herron J., Boland T., Fleming C., Egan M. (2024). Assessing the dry matter intake and enteric methane emissions of pre-partum dairy cows offered grass clover or grass-only silage from two different silage systems. Front. Anim. Sci..

[70] Min B.-R., Lee S., Jung H., Miller D.N., Chen R. (2022). Enteric Methane Emissions and Animal Performance in Dairy and Beef Cattle Production: Strategies, Opportunities, and Impact of Reducing Emissions. Animals.

[71] de Azevedo E.B., Savian J.V., do Amaral G.A., de David D.B., Gere J.I., Kohmann M.M., Bremm C., Jochims F., Zubieta A.S., Gonda H.L. (2021). Feed intake, methane yield, and efficiency of utilization of energy and nitrogen by sheep fed tropical grasses. Trop. Anim. Health Prod..

[72] Van Amburgh M.E., Russomanno K.L., Higgs R.A., Chase L.E. (2019). Invited review: Modifications to the Cornell Net Carbohydrate and Protein System related to environmental issues—Capability to evaluate nitrogen and phosphorus excretion and enteric carbon dioxide and methane emissions at the animal level. Appl. Anim. Sci..

[73] Wang Y., Song W., Wang Q., Yang F., Yan Z. (2024). Predicting enteric methane emissions from dairy and beef cattle using nutrient composition and intake variables. Animals.

[74] Niu M., Kebreab E., Hristov A.N., Oh J., Arndt C., Bannink A., Bayat A.R., Brito A.F., Boland T., Casper D. (2018). Prediction of enteric methane production, yield, and intensity in dairy cattle using an intercontinental database. Glob. Change Biol..

[75] Ornelas L.T.C., Silva D.C., Tomich T.R., Campos M.M., Machado F.S., Ferreira A.L., Maurício R.M., Pereira L.G.R. (2019). Differences in methane production, yield and intensity and its effects on metabolism of dairy heifers. Sci. Total Environ..

[76] Hayes B.J., Lewin H.A., Goddard M.E. (2013). The future of livestock breeding: Genomic selection for efficiency, reduced emissions intensity, and adaptation. Trends Genet..

[77] Grouven H. (1859). Vorträge über Agricultur-Chemie mit besonderer Rücksicht auf Thier- und Pflanzen-Physiologie.

[78] Henneberg W. (1870). Neue Beiträge zur Begründung Einer Rationellen Fütterung der Wiederkäner.

[79] Cabart M. (1838). Description de la caisse du calorimètre de M. Dulong. C. R. Acad. Sci..

[80] Kellner O., Köhler A. (1900). Untersuchungen über den Stoff- und Energie-Umsatz des erwachsenen Rindes bei Erhaltungs- und Produktionsfutter. Landwirtsch. Versuchsstat..

[81] Armsby H.P. (1903). The Principles of Animal Nutrition: With Special Reference to the Nutrition of Farm Animals.

[82] Oikawa K., Terada F., Kurihara M., Suzuki T., Nonaka I., Hosoda K., Kamiya Y., Roh S., Haga S. (2025). Methane emission prediction models for lactating cows based on feed intake, body weight, and milk yield and composition: Variable methane conversion factor-based approach. J. Dairy Sci..

[83] Jaurena G., Cantet J.M., Arroquy J.I., Palladino R.A., Wawrzkiewicz M., Colombatto D. (2015). Prediction of the Ym factor for livestock from on-farm accessible data. Livest. Sci..

[84] Liu Z., Liu Y., Shi X., Wang J., Murphy J.P., Maghirang R. (2017). Enteric methane conversion factor for dairy and beef cattle: Effects of feed digestibility and intake level. Trans. ASABE.

[85] Morrow B. (2021). Proposed Revision to the Cattle Methane Yield.

[86] Wang W.C., Yung Y.L., Lacis A.A., Mo T., Hansen J.E. (1976). Greenhouse effects due to man-made perturbations of trace gases. Science.

[87] Intergovernmental Panel on Climate Change (IPCC) (2019). Chapter 10: Emissions from Livestock and Manure Management. 2019 Refinement to the 2006 IPCC Guidelines for National Greenhouse Gas Inventories.

[88] Villanueva C., Ibrahim M., Castillo C. (2023). Enteric Methane Emissions in Dairy Cows with Different Genetic Groups in the Humid Tropics of Costa Rica. Animals.

[89] Volden H., Niu P., Prestløkken E. (2023). Models to Predict Enteric Methane Emission from Dairy Cows to Be Used in the Norwegian Inventory Calculations.

[90] Hristov A.N., Oh J., Giallongo F., Frederick T.W., Harper M.T., Weeks H.L., Branco A.F., Moate P.J., Deighton M.H., Williams S.R.O. (2015). An inhibitor persistently decreased enteric methane emission from dairy cows with no negative effect on milk production. Proc. Natl. Acad. Sci. USA.

[91] Hammond K.J., Crompton L.A., Bannink A., Dijkstra J., Yáñez-Ruiz D.R., O’Kiely P., Kebreab E., Eugène M., Yu Z., Shingfield K.J. (2016). Review of current in vivo measurement techniques for quantifying enteric methane emission from ruminants. Anim. Feed Sci. Technol..

[92] Johnson K.A., Huyler M.T., Westberg H.H., Lamb B.K., Zimmerman P. (1994). Measurement of methane emissions from ruminant livestock using a sulfur hexafluoride tracer technique. Environ. Sci. Technol..

[93] Storm I.M.L.D., Hellwing A.L.F., Nielsen N.I., Madsen J. (2012). Methods for measuring and estimating methane emission from ruminants. Animals.

[94] Garnsworthy P.C., Craigon J., Hernandez-Medrano J.H., Saunders N. (2012). On-farm methane measurements during milking correlate with total methane production by individual dairy cows. J. Dairy Sci..

[95] Yousef M.K., Johnson H.D. (1965). Helmet for continuous sampling of exhaled air of cattle. J. Dairy Sci..

[96] Burnheim K., Hughes K.J., Evans D.L., Raidal S.L. (2016). Reliability of breath-by-breath spirometry and relative flow-time indices for pulmonary function testing in horses. BMC Vet. Res..

[97] Fernández C.J., López M.D.C., Lachica M. (2012). Description and function of a mobile open-circuit respirometry system to measure gas exchange in small ruminants. Anim. Feed Sci. Technol..

[98] Schaller G.C., Harrison M.T. (2025). Developing accurate, cost-effective, practicable sensors for real-time determination of enteric methane emissions. Chem. Eng. J..

